# Investigation of neurophysiologic and functional connectivity changes following glioma resection using magnetoencephalography

**DOI:** 10.1093/noajnl/vdad091

**Published:** 2023-07-21

**Authors:** Nardin Samuel, Irene E Harmsen, Mandy Yi Rong Ding, Can Sarica, Artur Vetkas, Christine Wong, Vanessa Lawton, Andrew Yang, Nathan C Rowland, Suneil K Kalia, Taufik Valiante, Richard Wennberg, Gelareh Zadeh, Paul Kongkham, Aristotelis Kalyvas, Andres M Lozano

**Affiliations:** Toronto Western Hospital, Division of Neurosurgery, University Health Network, Toronto, Ontario, Canada; Toronto Western Hospital, Division of Neurosurgery, University Health Network, Toronto, Ontario, Canada; Mitchell Goldhar MEG Unit, University Health Network, Toronto, Canada; Krembil Research Institute, University Health Network, Toronto, Ontario, Canada; Toronto Western Hospital, Division of Neurosurgery, University Health Network, Toronto, Ontario, Canada; Toronto Western Hospital, Division of Neurosurgery, University Health Network, Toronto, Ontario, Canada; Toronto Western Hospital, Division of Neurosurgery, University Health Network, Toronto, Ontario, Canada; Toronto Western Hospital, Division of Neurosurgery, University Health Network, Toronto, Ontario, Canada; Department of Neurosurgery, Medical University of South Carolina, Charleston, South Carolina, USA; Department of Neurosurgery, Medical University of South Carolina, Charleston, South Carolina, USA; Murray Center for Research on Parkinson’s Disease and Related Disorders, Medical University of South Carolina, Charleston, South Carolina, USA; Toronto Western Hospital, Division of Neurosurgery, University Health Network, Toronto, Ontario, Canada; Krembil Research Institute, University Health Network, Toronto, Ontario, Canada; Toronto Western Hospital, Division of Neurosurgery, University Health Network, Toronto, Ontario, Canada; Mitchell Goldhar MEG Unit, University Health Network, Toronto, Canada; Toronto Western Hospital, Division of Neurology, University Health Network, Toronto, Ontario, Canada; Toronto Western Hospital, Division of Neurosurgery, University Health Network, Toronto, Ontario, Canada; Krembil Research Institute, University Health Network, Toronto, Ontario, Canada; Toronto Western Hospital, Division of Neurosurgery, University Health Network, Toronto, Ontario, Canada; Krembil Research Institute, University Health Network, Toronto, Ontario, Canada; Toronto Western Hospital, Division of Neurosurgery, University Health Network, Toronto, Ontario, Canada; Toronto Western Hospital, Division of Neurosurgery, University Health Network, Toronto, Ontario, Canada; Krembil Research Institute, University Health Network, Toronto, Ontario, Canada

**Keywords:** functional connectivity, glioma, magnetoencephalography, neural networks

## Abstract

**Background:**

In patients with glioma, clinical manifestations of neural network disruption include behavioral changes, cognitive decline, and seizures. However, the extent of network recovery following surgery remains unclear. The aim of this study was to characterize the neurophysiologic and functional connectivity changes following glioma surgery using magnetoencephalography (MEG).

**Methods:**

Ten patients with newly diagnosed intra-axial brain tumors undergoing surgical resection were enrolled in the study and completed at least two MEG recordings (pre-operative and immediate post-operative). An additional post-operative recording 6–8 weeks following surgery was obtained for six patients. Resting-state MEG recordings from 28 healthy controls were used for network-based comparisons. MEG data processing involved artifact suppression, high-pass filtering, and source localization. Functional connectivity between parcellated brain regions was estimated using coherence values from 116 virtual channels. Statistical analysis involved standard parametric tests.

**Results:**

Distinct alterations in spectral power following tumor resection were observed, with at least three frequency bands affected across all study subjects. Tumor location-related changes were observed in specific frequency bands unique to each patient. Recovery of regional functional connectivity occurred following glioma resection, as determined by local coherence normalization. Changes in inter-regional functional connectivity were mapped across the brain, with comparable changes in low to mid gamma-associated functional connectivity noted in four patients.

**Conclusion:**

Our findings provide a framework for future studies to examine other network changes in glioma patients. We demonstrate an intrinsic capacity for neural network regeneration in the post-operative setting. Further work should be aimed at correlating neurophysiologic changes with individual patients’ clinical outcomes.

**Key Points**

1. Distinct alterations in regional and functional connectivity persist for at least 6–8 weeks following glioma resection.
2. There is an intrinsic capacity for regional functional connectivity following glioma resection.
3. Comparable changes in inter-regional functional connectivity were noted across patients following glioma resection.

**Importance of the Study**

With the emergence of connectomics, the importance of a personalized, meta-network perspective to surgical planning is being recognized. Currently, the application of connectomics in surgery is limited by a lack of understanding of network recovery following surgery. Traditionally, surgical selection for gliomas was based on tumor topography. As such, our study is timely because it demonstrates the restoration of regional functional connectivity following glioma surgery and provides a framework for assessing the functional impact of tumor resection. Our study will also guide future studies on applying imaging and neurophysiologic tools to assess patients’ post-operative outcomes.

Previous investigations in glioma patients have found that neural network changes, including increased global brain network clustering and decreased global synchronizability, relate to poorer cognition and epilepsy.^1,2^ Network dysfunction has also been associated with behavioral, cognitive, and emotional impairment in glioma patients before and after surgery, reducing patients’ quality of life.^3–6^ Moreover, tumor-induced neural reorganization of language has been detected in patients with brain tumors in the language-dominant hemisphere.^7^ Collectively, the literature has provided strong support that gliomas affect and alter existing neural networks. However, there remains a gap in our understanding of how neural networks respond following glioma resection.

The technologies currently available to investigate human brain activity vary widely along the temporospatial continuum. Electrophysiological modalities such as electroencephalography (EEG) and electrocorticography (ECoG), while capable of measuring subsecond changes in neural activity, are limited by spatial resolution and invasiveness, respectively. Functional magnetic resonance imaging (fMRI) can obtain high spatial resolution but is limited in resolving subsecond neural activation. Magnetoencephalography (MEG) is a recording modality that ideally bridges the desired temporospatial continuum.^8^ MEG is similar to EEG and ECoG in that it localizes neural activity in the brain on the order of milliseconds. However, MEG is fundamentally different because it does not detect the voltages generated by neural currents but rather from the resultant magnetic fields.^8,9^ The skull does not attenuate the magnetic fields associated with the brain’s electric currents, which enables MEG to detect higher-frequency electromagnetic oscillations more reliably. These changes are typically too low in amplitude to be detected by conventional EEG. Compared to EEG, MEG is relatively insensitive to the electrical muscle activity of the scalp, further increasing the ability of MEG to measure high-frequency neural activity. Modern MEG devices can also non-invasively detect the brain’s magnetic fields over the entire cortical envelope. In addition, MEG is most sensitive to activity originating in sulci due to its detection of tangential components in the head.^10^ This is advantageous in neurosurgical patients where cortical disruptions following tumor resection can be mostly expected in sulci instead of superficial gyri (ECoG measures activity here and is a limitation of its use). These characteristics support using MEG over other technologies to measure neuronal activity, particularly in patients undergoing a neurosurgical procedure.

In the present study, MEG was used to determine the relationship between intra-axial tumors and cortical physiology by exploring changes in brain activity before and after surgery. This investigation aimed to map the neurophysiologic basis of network recovery following resection, with the hypothesis that there is an intrinsic capacity for functional reorganization in the post-operative setting. Our findings provide insight into the effect of gliomas on neural networks and a basis for understanding the plastic potential of the brain in the context of glioma resection.

## Methods

### Study Subjects, Experimental Procedure, and Surgical Resection

Ten patients with radiographically suspected glioma (4 females, age range: 25–81 years, mean age 55.3 years, SD: ±18.4 years; Table 1) were recruited from Toronto Western Hospital, University Health Network (UHN). Patients were screened by a clinical staff member of the Oncology service and referred to the study. Inclusion criteria included: (1) English-speaking patients above 18 years of age, (2) patients with a newly diagnosed intra-axial brain tumor with radiographic features suggestive of an invasive glioma or high-grade glioma, and (3) patients capable of providing informed consent. Exclusion criteria included: (1) participants with underlying conditions that would preclude lying supine in the MEG unit, (2) patients with co-morbid neurological or psychiatric disorders, and (3) participants unable to lie still during the recording session without discomfort, (4) patients with histologically confirmed WHO grade 1 tumors. The study was approved by the UHN (Toronto, Canada) Research Ethics Board (CAPCR 15-9758). Resting-state MEG data obtained from 28 healthy controls were used for network-based comparisons (13 females, age range: 17–79 years, mean age 37.8, SD: ± 3.7 years).

**Table 1.** Patient demographics, clinical information. and tumor characteristics.

ID	Age	Sex	Tumor location	Extent of resection (EOR)	Clinical data			Pathology					
					Epilepsy features	Medications	Adjuvant therapy	Tissue diagnosis	IDH mutation status	MGMT hypermethylation status	WHO grade	TP53 status	1p/19q codeletion status
1	37	F	Left temporoparietal/insular	Gross total	Focal seizures on presentation (acute onset confusion, speech deficits), ongoing post-operative	Keppra 2000mg po qam and 1500mg po qpm (lacosamide dexa-mathesaone discontinued)	TMZ + radiation	Oligodendroglioma	Mutant	*	2	Wild type	Positive
2	41	M	Left temporal	Subtotal	GTC seizure at presentation, no seizures thereafter	Keppra 1250mg po bid	TMZ + radiation	Astrocytoma	Mutant	Negative	2	Mutant	Negativ e
3	74	M	Right temporal	Gross total	Olfactory hallucinations	Keppra 500 mg po bid, dexamethasone 4 mg po daily	TMZ + radiation Beva-cizumab	High-grade glioma	Wild type	Negative	3	Wild type	*
4	25	M	Right parietooccipital	Gross total	GTC on presentation, no seizures thereafter	Keppra 1000mg po bid	TMZ + radiation	Oligodendroglioma	Mutant	*	3	*	Positive
5	44	F	Left parietooccipital	Gross total	History of seizures in childhood; GTC on presentation, no seizures thereafter	—	None	Pilocytic astrocytoma	Wild type	Negative	1	Wild type	*
6	81	M	Right frontotemporal	Partial	None	—	None (patient deceased prior to initiating adjuvant therapy)	GBM	Wild type	Positive	4	Wild type	*
7	72	F	Left parietooccipital	Subtotal	None	-	TMZ + radiation	GBM	Wild type	Negative	4	Wild type	*
8	51	M	Right temporal	Gross total	GTC on presentation, no seizures thereafter	Keppra 750 mg po bid, Dexamethasone 4 mg po daily	TMZ + radiation	GBM	Wild type	Positive	4	Wild type	*
9	63	M	Left frontoparietal	Gross total	No seizure at presentation, new onset seizure 12 mos post-op (12X GTC)	Phenytoin 100 mg po tid	TMZ + radiationbeva-cizumab	GBM	Wild type	Negative	4	Wild type	*
10	65	F	Right temporal	Gross total	Olfactory seizures at presentation	Keppra 100 mg po daily	TMZ + radiation	GBM	Wild type	Negative	4	Wild type	*

Asterisk (*) denotes unavailable information. F, female; GBM, glioblastoma multiforme; IDH, isocitrate dehydrogenase; M, male; MGMT, O(6)-methylguanine-DNA methyltransferase; WHO, World Health Organization; GTC, generalized tonic-clonic seizure; Keppra (levetiracetam); po, per os (by mouth); bid, twice daily; tid, three times daily. TMZ, temozolamide.

Written informed consent was obtained from each subject, and all experimental procedures were performed in accordance with the Declaration of Helsinki. Patients were enrolled to undergo at least two MEG recordings (pre-operative and <48 hours post-operative). When scheduling permitted, subjects underwent an additional post-operative recording 6–8 weeks following surgery (Subjects 1, 2, 4, 5, 7, and 9). All patients underwent surgical resection of their tumors with intra-operative neuro-navigation following standard surgical procedures. There were no modifications to the clinical management of these patients related to the present study. All patients tolerated the procedures well; no intra-operative or post-operative complications occurred.

### Magnetoencephalography Acquisition

MEG recordings were conducted using a 306-channel MEG system (Elekta Neuromag TRIUX, Helsinki, Finland) at a sampling rate of 1000 Hz with an online bandpass filter between 0.1 and 330 Hz. Recording occurred while participants were in a supine position with their eyes closed. Participants underwent triplicate measurements, two minutes in duration each. All measurements were included in the analysis.

### Magnetoencephalography Data Processing and Analysis

Artifact suppression in the MEG data was performed using the temporal signal space separation (tSSS) algorithm implemented within the Neuromag MaxFilter system (software version 2.2.12, Elekta, Helsinki, Finland)^11–13^ with the default 10-second time window and subspace correlation limit of 0.980. Gradiometer data was processed and analyzed in Matlab (The Mathworks, Natick, MA, USA) using the FieldTrip toolbox^14^ and custom-made scripts. Data were epoched into 1-second trials, and a high-pass filter of 1 Hz was applied to remove drifts. Filters were also applied at 60, 120, and 180 Hz to remove power line noise and harmonics. All raw data were visually inspected, and segments with signal dropouts or artifacts were discarded.

For source power and coherence analyses, the time-series MEG data were transformed to the frequency domain using a multi-taper method fast Fourier transform. Power spectra were then generated using a multi-taper frequency transformation^15^ and discrete prolate spheroidal sequences. To estimate spectral power for specific brain regions, sources were localized using dynamic imaging of coherent sources (DICS).^16^ The Automated Anatomical Labeling (AAL) atlas was used to parcellate the single-subject Montreal Neurological Institute (MNI)-space template brain, derived from participants’ structural T1-weighted MRI scans obtained pre-operatively, such that absolute power estimates were generated for 116 brain regions.^17,18^ Frequencies were averaged into delta (1-3 Hz), theta (4-8 Hz), alpha (8-12 Hz), beta (13-29 Hz), low gamma (30-50 Hz), mid gamma (50-70 Hz), and broadband gamma (70-170 Hz) bands. Regions of spectral power desynchronization were depicted on the MNI ICBM152 non-linear 6th Generation Symmetric Average Brain Stereotaxic Registration Model using FSL software (FMRIB Software Library v6.0).^19^

We used *ft_freqstatistics* and *ft_clusterplot* for the visualization of significant sensor-level regions. DICS beamformer source power results were visualized using 3D surface brains. In addition, functional connectivity between AAL-labeled brain regions was estimated using coherence values from 116 virtual channels.^19–22^ Separate matrices were generated for each condition and consisted of 116 × 116 brain region pairs. For comparative purposes, source regions were clustered into eight major bilateral brain regions (frontal, sensorimotor, basal ganglia, limbic, parietal, temporal, occipital, and cerebellar) and midline vermis. Local functional connectivity was estimated by averaging coherence for all brain region pairs within each major brain region. Inter-regional (between region of interest (ROI)) connectivity was estimated by averaging coherence for all brain region pairs between two ROIs and included interhemispheric (left vs. right) and intra-hemispheric (left vs. left and right vs. right) comparisons.

### Statistical Analysis

Parametric statistics were used for MEG data as it is “super-averaged” (e.g. sensorimotor cortical beta source power is an average of its anatomical parcel, 17 frequencies, and 120 epochs).^23–25^ Statistical analyses were conducted using Matlab (Mathworks, Natick, MA, USA, http://www.mathworks.com).

## Results

### Study Subjects and Tumor Profiles

**Figure 1.**
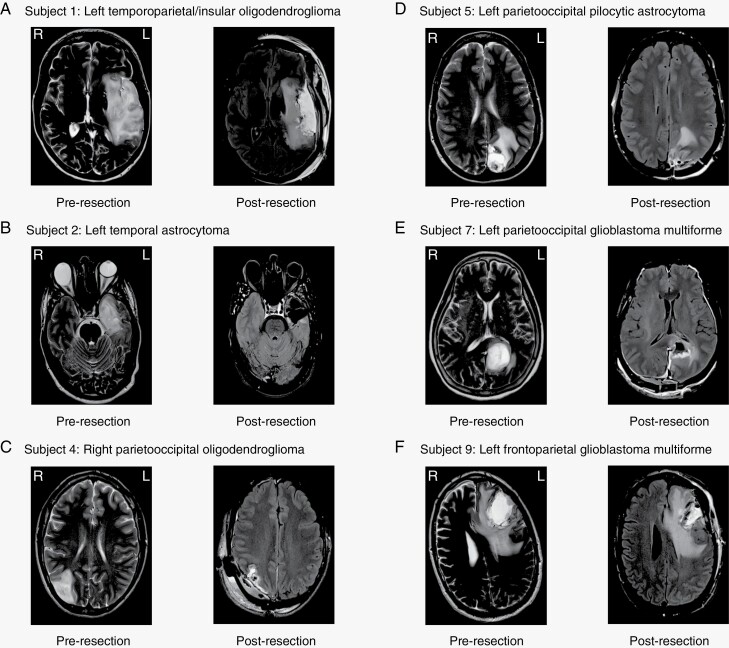
Pre- and post-resection neuroimaging. (**A-J**) Axial views of T2-weighted magnetic resonance imaging (MRI) (pre-) and T2-FLAIR (post) sequences for each study subject with delayed post-resection MEG recordings. Images were acquired before surgery and <48 hours following tumor resection. Note that the same imaging sequences are displayed for consistency. In some cases of high-grade tumors, edema, and post-operative mass effect may be more apparent.

Ten patients with radiographically suspected glioma (subsequently confirmed histologically) participated in the study and underwent at least two sessions of MEG recordings (pre-operative and <48 hours post-operative; Table 1). The radiographic features and distribution of the anatomical location of tumors are shown in Figure 1 for patients who completed an additional MEG recording 6-8 weeks post-operatively.

### Distinct Alterations in Spectral Power Persist Following Tumor Resection

**Figure 2.**
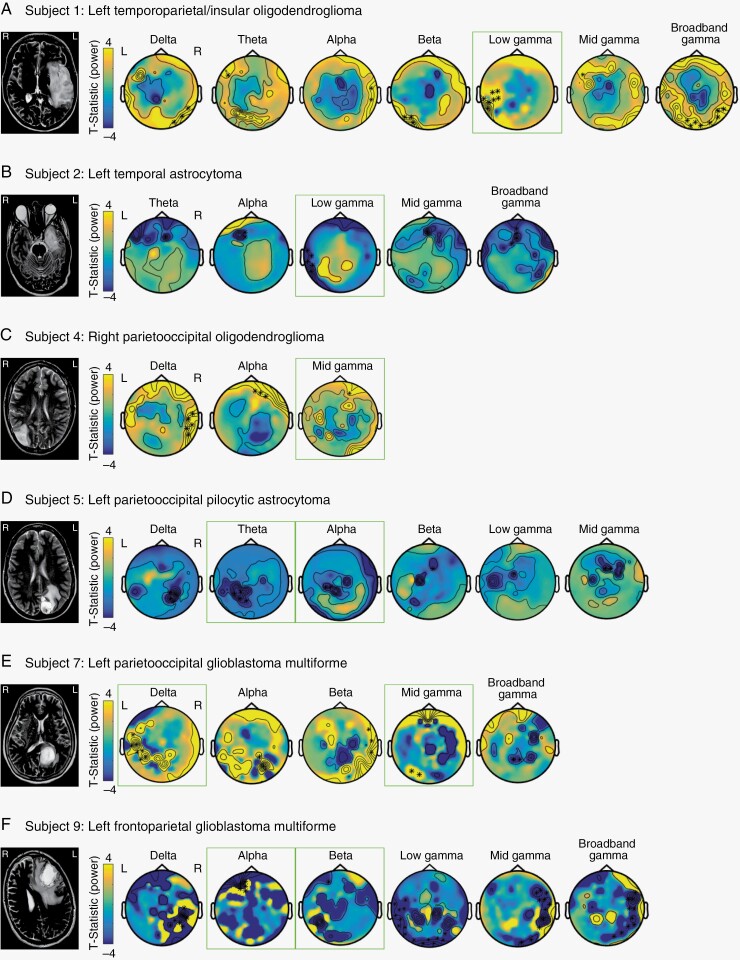
Changes in MEG-derived spectral power following tumor resection. (**A–F**) Topographical plots show sensor-level cluster-based permutation testing for delayed post-resection brain activity compared to pre-resection brain activity. Plots are shown only for frequency bands with a statistically significant change in spectral power, Bonferroni-corrected (*n* = 7 multiple comparisons for frequency bands), *P* < .0071. Green boxes highlight significant frequencies in the anatomical vicinity of the tumor. Yellow (positive *T*-statistic values) represent an increase in spectral power post-resection. Blue (negative *T*-statistic values) represent a decrease in spectral power post-resection. Frequency bands: delta (1–3 Hz), theta (4–8 Hz), alpha (8–12 Hz), beta (13–29 Hz), low gamma (30–50 Hz), mid gamma (50–70 Hz), and broadband gamma (70–170 Hz). MR images (*left*) show the anatomical orientation of tumor location.

To investigate lasting changes in MEG-derived spectral power following surgery, we compared baseline (pre-operative) versus delayed post-operative (6–8 weeks post-resection) MEG recordings. Cluster-based permutation testing on sensor-level data was performed to compare delayed post-resection measures and pre-resection brain activity. Changes were noted in at least three frequency bands across all study subjects (Figure 2). Moreover, spectral power-related changes in the anatomical vicinity of the individual patients’ tumors were identified for specific frequency bands unique to each patient. In particular, tumor location-related changes in low-mid gamma frequencies (30–70 Hz) were found in four patients (Subjects 1, 2, 4, and 7), and alpha frequency (8–12 Hz) changes were found in two patients (Subjects 5 and 9; Figure 2).

### Recovery of Regional Functional Connectivity Occurs Following Glioma Resection

**Figure 3.**
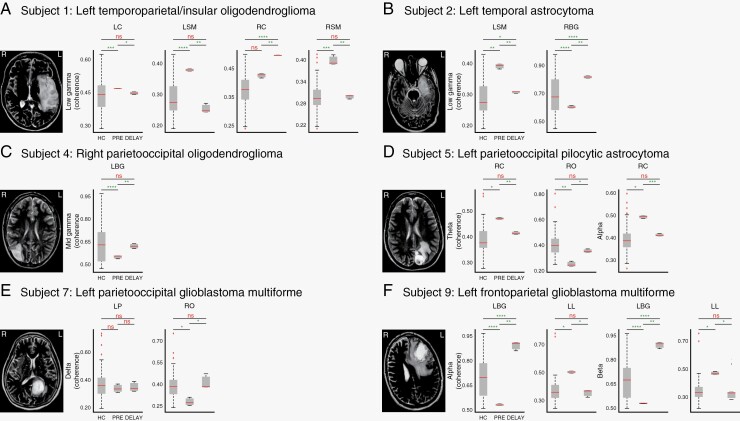
Recovery of regional functional connectivity following tumor resection. (**A–F**) Boxplots comparing local connectivity (regional coherence) between healthy controls (HC, *n* = 28 [56 measurements]) and glioma patients pre-resection and delayed post-resection (triplicate measurements for all conditions). Frequencies of interest are determined from significant changes in spectral power following tumor resection specific to tumor location (refer to Figure 2, green boxes). Plots are shown for brain regions with significant changes in coherence between groups, showing a recovery of abnormally low or high pre-resection coherence to levels found in healthy controls. Regional functional connectivity was estimated by averaging coherence for all brain region pairs within each of the major brain regions. Brain regions: left (L) and right (R) frontal (F), sensorimotor (SM), parietal (P), occipital (O), limbic (L), basal ganglia (BG), temporal (T), and cerebellar (C). Frequency bands: delta (1–3 Hz), theta (4–8 Hz), alpha (8–12 Hz), beta (13–29 Hz), low gamma (30–50 Hz), mid gamma (50–70 Hz), and broadband gamma (70–170 Hz). ns, not significant; *P < .05; **P < .01; ***P < .001; ****P < .0001. MR images (*left*) show the anatomical orientation of tumor location.

Next, we explored the extent to which regional neural networks recover following surgery. To investigate this, we compared the functional connectivity of regional networks in healthy controls to those in glioma patients. Specifically, we assessed the “normalization” of these networks in the delayed post-resection measurements relative to pre-operative recordings (Figure 3). We also evaluated changes in spectral power and regional functional connectivity immediately following tumor resection (<48 hours post-operative) to ascertain transient post-surgical changes in neuronal pathways in all ten study subjects (Supplementary Figure 1).

These findings provide a roadmap for the recovery of distinct regional connectivity patterns that appear to normalize or trend toward levels of healthy controls in a delayed fashion following surgery (Figure 3). These patterns differ in regions and frequency band patterns across individuals, suggesting patient-specific and tumor-specific changes. We also observed significant comparable changes in low-mid gamma frequency-associated functional connectivity in four study subjects.

### Inter-regional Functional Connectivity Changes Following Glioma Resection

**Figure 4.**
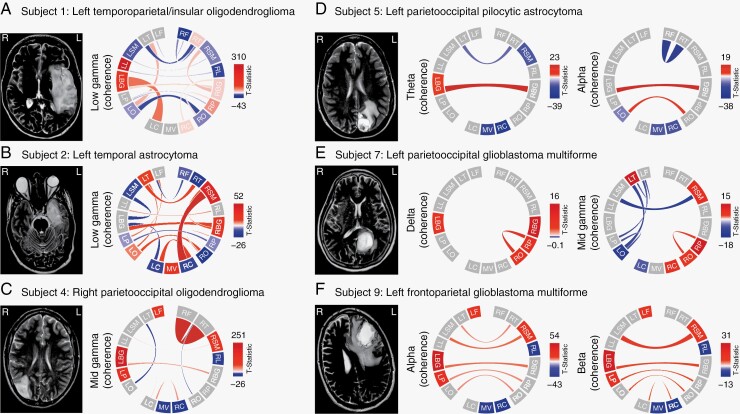
Changes in inter-regional functional connectivity following tumor resection. (**A–F**) Functional connectivity maps (connectograms) depict changes in coherence for delayed post-resection relative to pre-resection brain activity. Frequencies of interest are determined from significant changes in spectral power following tumor resection specific to tumor location (refer to Figure 2, green boxes). Intra-regional functional connectivity was estimated by averaging coherence for all brain region pairs within each major brain region. Inter-regional (between region-of-interest (ROI)) connectivity was estimated by averaging coherence for all brain region pairs between two ROIs and included interhemispheric (i.e. left vs. right) and intrahemispheric (i.e. left vs. left, right vs. right) comparisons. Red (positive *T*-statistic values) represent a significant increase in coherence post-resection (“functional coupling”). Blue (negative *T*-statistic values) represent a significant decrease in coherence post-resection (“decoupling”). Brain regions: left (L) and right (R) frontal (F), sensorimotor (SM), parietal (P), occipital (O), limbic (L), basal ganglia (BG), temporal (T), and cerebellar (C), and midline vermis (MV). Frequency bands: delta (1–3 Hz), theta (4–8 Hz), alpha (8–12 Hz), beta (13–29 Hz), low gamma (30–50 Hz), mid gamma (50–70 Hz), and broadband gamma (70–170 Hz). MR images (*left*) show the anatomical orientation of tumor location.

Next, we aimed to determine the impact of glioma resection on global functional connectivity patterns across the brain. We constructed functional connectivity maps (connectograms) to illustrate the delayed post-operative global patterns in functional connectivity for each patient (Figure 4). Notably, patterns detected in individual patients demonstrate a unique “fingerprint” of tumor-associated connectivity changes. These findings again demonstrate distinct patterns, with recurrent changes in low-mid gamma-associated functional connectivity in four patients. This global analysis is essential as it provides a framework for better understanding the local and remote connectivity patterns that are altered or recovered post-operatively.

### Baseline and Post-operative Changes in Functional Connectivity Correlate With Clinical Features

Lastly, we determined whether the recovery of regional functional connectivity occurring post-resection correlates with clinical features and outcomes outlined in Table 1. Using Pearson’s linear regression, we found that absolute changes in functional connectivity 6–8 weeks post-operatively (delayed post-operative coherence minus pre-operative coherence) correlated with IDH mutation status (*R* = −0.30, *P* = .041, data not shown) and WHO tumor grade (*R* = 0.47, *P* = 8.64e−4, data not shown). Wild-type IDH mutation status was associated with greater absolute changes in functional connectivity post-operatively, possibly suggesting that patients with wild-type tumors have a greater capacity for functional recovery of neural networks than patients with IDH mutant tumors. High-grade tumors were associated with greater absolute changes in functional connectivity post-operatively, perhaps due to increased aberrant connectivity that is “normalized” following resection. Relatedly, the occurrence of seizures post-operatively was associated with greater absolute changes in functional connectivity post-operatively (*R* = 0.36, *P* = .012, data not shown). Seizures occurring post-resection may result from increased aberrant connectivity that is “normalized” following resection. However, tumor grade may confound this finding since seizure activity post-resection occurred in patients with higher tumor grades. Notably, the extent of resection was not associated with absolute changes in functional connectivity post-operatively (*R* = 0.11, *P* = .472, data not shown).

We also examined our entire cohort of tumor patients (*n* = 10) to determine if baseline neural network dysfunction (pre-resection regional coherence) correlates with different grades of glioma. Increasing WHO grade positively correlates with regional coherence (*R* = 0.48, *P* = 7.10e−3, data not shown). This means that high-grade tumors (WHO grades 3–4) have higher regional coherence at baseline (pre-resection) compared to low-grade tumors (WHO grades 1–2). As such, cortical connectivity may be an important biomarker to consider for diagnosis and prognostication of tumors before undergoing surgical biopsy or resection.

## Discussion

The present study provides a personalized neurophysiologic assessment of global and regional functional connectivity in glioma. We demonstrate that regional functional connectivity can be restored after glioma resection and that glioma resection alters global functional connectivity. These neuroplastic changes occur uniquely and should be assessed at an individual patient level. There has been increasing recognition of the complex interplay between normal brain function and glioma growth and progression.^26^ Several studies have supported the burgeoning field of “cancer neuroscience,” which is grounded in the finding that gliomas integrate within normal neural networks, and promote the formation of aberrant functional networks.^27^ Moreover, MEG studies demonstrate that gliomas tend to occur in regions with intrinsically higher activity levels.^28^ MEG has also been used to highlight the widespread changes in global network clustering in glioma and identify the dependence of these changes on tumor IDH mutation status.^1,29,30^ Lastly, MEG-derived signatures of functional synchronization have been found to correlate with tumor histology.^31^ This may partly explain the clinical manifestations of cognitive deficits and epileptic seizures that commonly occur in patients with glioma. Studies have demonstrated that lesion histology correlates with differences in functional networks in glioma patients with epilepsy.^2^

While the results from the present study support these findings, we go on to show that changes in regional functional connectivity occur at the tumor location post-resection. In particular, changes in local coherence in the low- and mid-gamma frequency range (30–70 Hz) were noted. Low- and mid-gamma frequency bands coordinate various cognitive processes such as perception, attention, and memory.^32^ In higher-order cortical regions, gamma activity is involved in attentional processes.^32^ Importantly, gamma oscillations have been associated with neural synchronization and communication between different brain regions. Our study further validated this with the findings of inter-regional alterations in gamma coherence. These results suggest that tumors appear to alter the underlying connectivity networks that control higher-order cognitive processes in the brain.

Our findings demonstrate coupling and decoupling patterns that cannot be solely attributed to the preservation/re-activation or disruption of known white matter connections between respective areas. Recent tractography and functional studies emphasize the dynamic nature of brain connectivity and the potential for reorganization.^33,34^ For instance, gliomas can directly damage white matter tracts and indirectly affect functional network organization.^35^ Additionally, gliomas can cause increased or decreased functional connectivity depending on tumor location and characteristics.^36,37^ These findings underscore the complex interplay between brain tumors and the brain’s intrinsic functional networks. In future studies, combining MEG with other imaging modalities, such as diffusion tensor imaging (DTI) and resting-state functional MRI (rs-fMRI), may elucidate mechanisms driving connectivity changes. This could provide insights for personalized brain tumor management and neurorehabilitation strategies.

The results from this study provide a framework for assessing the functional impact of tumor resection and can be translated to the use of other modalities such as EEG and fMRI. The results are intended to provide an individualized assessment of neurophysiologic changes imparted by tumors and following their resection. Previous studies aimed at assessing tumor volumes with MEG-derived metrics of brain activity have failed to identify any recurrent patterns due to the approach of data aggregation.^38^ Rather than aggregating and analyzing cohort-level data, a personalized and patient-specific approach to understanding network-level changes is essential due to marked inter-individual variability in tumor size, location, histopathology, and the individual patient’s baseline functional connectivity patterns. We found that pre-operative functional connectivity correlates with glioma WHO grade, which aligns with other studies suggesting differences between baseline neural network dysfunction for patients with different grades of glioma.^2,31^ Moreover, the capacity for functional recovery of neural networks may correlate with these factors and the extent of tumor infiltration. Relating the integrity and recovery of neural networks with clinical status may have utility in prognostication, as well as future research into neuromodulation strategies to augment post-operative rehabilitation.^39^ Although we explored associations between post-operative changes in functional connectivity and clinical features and outcomes (ie, IDH mutation status, WHO grade, post-operative seizure occurrence), our results cannot be deemed conclusive due to the study’s sample size. Traditionally, surgical selection and planning for gliomas were based on tumor topography. With the emergence of connectomics, there has been increasing recognition of the importance of a meta-network perspective to surgical planning.^40^

Some study limitations should be noted. Firstly, not all patients could complete multiple serial MEG recordings, highlighting the challenges of obtaining consistent data on this patient population. In addition, the timing of our delayed post-operative measurements may be confounded by residual acute post-operative changes or new disease-associated symptoms such as inflammation, newly developed seizures, or adjuvant therapies. Secondly, the specific connectivity patterns could not be correlated with cognitive or psychiatric outcomes since formal neuropsychological testing was not performed. Finally, while MEG is a gold standard tool for studying neural oscillations, it is not widely accessible. As such, more work is needed to validate alternate imaging and neurophysiologic approaches to refine protocols for assessing neuroplasticity at an individual level.

## Conclusion

This study used serial MEG recordings and compared pre- and post-operative brain states to explore changes in brain activity following glioma resection. This work demonstrates that regional functional connectivity can be restored following glioma surgery and provides a framework for assessing the functional impact of tumor resection. These findings can guide further studies on applying imaging and neurophysiologic tools to assess patients’ post-operative outcomes. Future work should be aimed at correlating these findings with individual clinical outcomes to adopt a personalized approach to the neurosurgical management of glioma.

## Supplementary Material

vdad091_suppl_Supplementary_Figure_S1

vdad091_suppl_Supplementary_Data

## Data Availability

Data supporting the findings of this study are available from the corresponding author, A.M.L., upon reasonable request.
