# Supplementary material for: Investigation of neurophysiologic and functional connectivity changes following glioma resection using magnetoencephalography
Source: Neurooncol Adv. 2023 Jul 21;5(1):vdad091. doi: 10.1093/noajnl/vdad091 (PMC10403751; doi:10.1093/noajnl/vdad091)
Supplement: vdad091_suppl_Supplementary_Data [file vdad091_suppl_supplementary_data.docx]

**Supplementary Figure 1. Concordant changes in spectral power and (intra)regional functional connectivity immediately following tumor resection.** **(A-J)** In the acute post-operative phase and specific to tumor location, spectral power increases are associated with increases in (intra)regional functional connectivity, and spectral power decreases are associated with decreases in (intra)regional functional connectivity. Topographical plots (*middle*) show sensor-level cluster-based permutation testing for immediate post-resection minus pre-resection brain activity. Plots are shown for frequency bands with a significant change in spectral power, denoted by a black asterisk (*), Bonferroni-corrected (n=7 multiple comparisons for frequency bands), p<0.0071. Yellow (positive T-statistic values) represent an increase in spectral power post-resection. Blue (negative T-statistic values) represent a decrease in spectral power post-resection. Boxplots (*right*) compare local coherence between pre-resection (Pre, n=3) and immediate post-resection (Immed, n=3) time points for each patient. (Intra)regional functional connectivity was estimated by averaging coherence for all brain region pairs within each major brain region. Brain regions: left (L) and right (R) frontal (F), sensorimotor (SM), parietal (P), occipital (O), limbic (L), basal ganglia (BG), temporal (T), and cerebellar (C). Frequency bands: delta (1-3 Hz), theta (4-8 Hz), alpha (8-12 Hz), beta (13-29 Hz), low gamma (30-50 Hz), mid gamma (50-70 Hz), and broadband gamma (70-170 Hz). ns, not significant; *, p<0.05; **, p<0.01; ***, p<0.001. MR images (*left*) show the anatomical orientation of tumor location.
